# BOLD signal simulation and fMRI quality control base on an active phantom: a preliminary study

**DOI:** 10.1007/s11517-020-02133-9

**Published:** 2020-02-08

**Authors:** Tiao Chen, Yue Zhao, Chuntao Jia, Zilong Yuan, Jianfeng Qiu

**Affiliations:** 1 Medical Engineering and Technology Research Center, Shandong First Medical University & Shandong Academy of Medical Sciences, Taian, 271016 China; 2 Department of Radiology, Hubei Cancer Hospital, Tongji Medical College, Huazhong University of Science and Technology, Wuhan, 430079 China; 3 Radiology Department, Shandong First Medical University & Shandong Academy of Medical Sciences, Medical Sciences, No. 619, Changcheng Road, Taian, 271016 China; 4 Shandong University of Science and Technology, Qingdao, 266590 China

**Keywords:** Phantom, BOLD, fMRI, Current simulation, Quality control

## Abstract

Blood-oxygen-level-dependent (BOLD) signal has been commonly used in functional magnetic resonance imaging (fMRI) to observe the activity in different areas of the brain or other organs. This signal is difficult to simulate, because its amplitude is nearly 1~3% and it is influenced by multiple factors. This study aimed to design and construct an active BOLD simulation phantom and test its stability and repeatability. The phantom consisted of two perpendicular loops. The BOLD signal was simulated by different stimuli generated by a regular periodic vibration current and transmission loops. Three scanners (Siemens skyra 3.0 T, Siemens verio 3.0 T, and GE signa HD 1.5 T) were used to test the stability and repeatability of the BOLD signal detection of the phantom. The percent signal change (PSC) was calculated for each stimulus. At baseline, the phantom exhibited stability, and the average signal variation was below 1% as revealed by the three scanners. The SNR of ROIs with different sizes were markedly high, being 2326.58 and 2389.24; and the ghosting ratio were 0.39% and 0.38%, and the stimuli detection efficiency for Siemens verio and Siemens skyra was 60% and 75%, respectively. The repeated scans of the same scanner for different stimuli were highly reproducible. In the three scanners, the PSC at the same location varied from nearly 1 to 3%. The areas activated on the phantom revealed by different scanners were comparatively consistent. The phantom designed for fMRI quantitative quality control displays good adaptability to different scanners and is easy to operate. It can reliably collect data by simple data processing.

**Graphical abstract:**
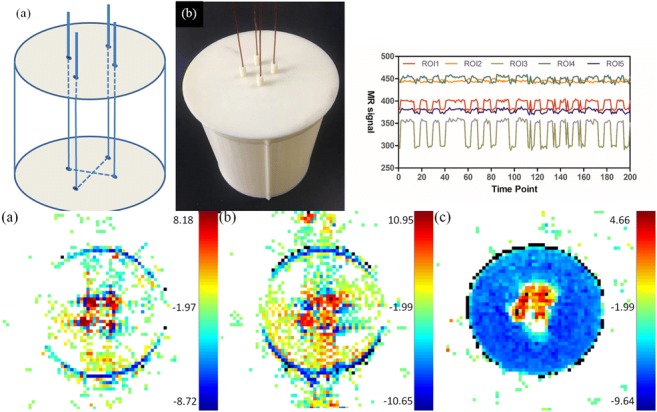
fMRI phantom testing process

fMRI phantom testing process

## Introduction

Functional magnetic resonance imaging (fMRI) is an effective tool for noninvasively studying the neurophysiological brain activity. The technique is based on the theory of blood-oxygen-level-dependent (BOLD). Through this technique, the hemodynamic response related to neuronal signal can be assessed by taking advantage of difference in magnetic susceptibility of deoxygenated and oxygenated blood. It has been established that the BOLD signal amplitude on the T2* image varies by approximately 1–3% [1, 2], and its signal peaks about 5-8s post stimulation before falling back to the baseline level almost at the same time [3]. This signal amplitude change is very small in relation to the imaging signal produced by the brain tissue itself. Consequently, in order to detect BOLD signals at good resolution, sufficient time and signal detection efficiency are needed. It should also be noted that multiple properties of the magnetic resonance (MR) scanner itself, including the magnetic field intensity, gradient, sequence design, and data processing protocol, can influence signal detection. In addition, during functional MR scanning, normal physiological processes like breathing, pulse, and head movement impact the BOLD signal and affect reproducibility [4, 5].

Collectively, these factors necessitate quality control (QC) analysis for fMRI. It is necessary to quantitatively establish the detection efficiency of the BOLD signal produced by MR system prior to fMRI analysis. Currently, there are two main types of phantoms used in quality control analysis of an MR system, i.e., the digital phantom and the physical phantom. These approaches are used to optimize image acquisition strategies, image post-processing, and image reconstruction algorithms and to compare the performance of differential imaging systems [6–8]. It should be noted that these phantoms do not account for the real (MR) system behavior including the noise introduced during the imaging process, such as noise from the MR power amplifier, preamplifier, transmit/receive, and digitization (A/D) error [9]. On the other hand, because the ground truth of the physical phantoms’ parameters is well established, acquired experimental data is regarded the gold standard [10]. The QC of MR systems is normally done by physicists and technicians using physical phantom as auxiliary tools for inspection. Compared to digital phantoms, physical functional MR phantoms are more effective. Physical phantoms are therefore better suited for objective testing and are typically interpreted by technologists and physicists to determine if the MR system meets clinical demands. However, conventional quality control phantom and scanning protocols, based on the American College of Radiology (ACR) standard and AAPM report, only evaluates spatial resolution, geometric distortion, layer thickness and accuracy, image uniformity, signal ghost ratio, and low contrast resolution of MRI imaging. Currently, detection sensitivity for BOLD-related signals cannot be calibrated.

Multiple studies have explored fMRI quality control methods. A study mimicking the T1 and T2 properties of the gray matter evaluated a range of static MR scanner features including SNR and noise but could not simulate task-related stimulation [11]. Another study used two liquids with slightly different T1, T2, and T2* to mimic the control and activation states of the brain and moved the “control part” and “activation part” manually to the center of the coil during the experiment [12], making the approach cumbersome. Current-induced magnetic field has also been used to change the local magnetic field (B_0_) near the conductor. This approach has the advantage that signal changes are easily synchronizable with fMRI experiments. However, the phantom used in this study had a small area for analysis, making it susceptible to magnetic field heterogeneity [13].

In a bid to limit the shortcomings highlighted above, we developed a BOLD signal simulation phantom, and applied it in the assessment of SNR, SFNR, and ghost signal ratio and carried out a block design experiment of adjustable stimulus size and duration. Furthermore, we evaluated the signal detection efficiency and percent signal change (PSC) using different frequently used scanners.

## Materials and methods

### Phantom properties

**Fig. 1:**
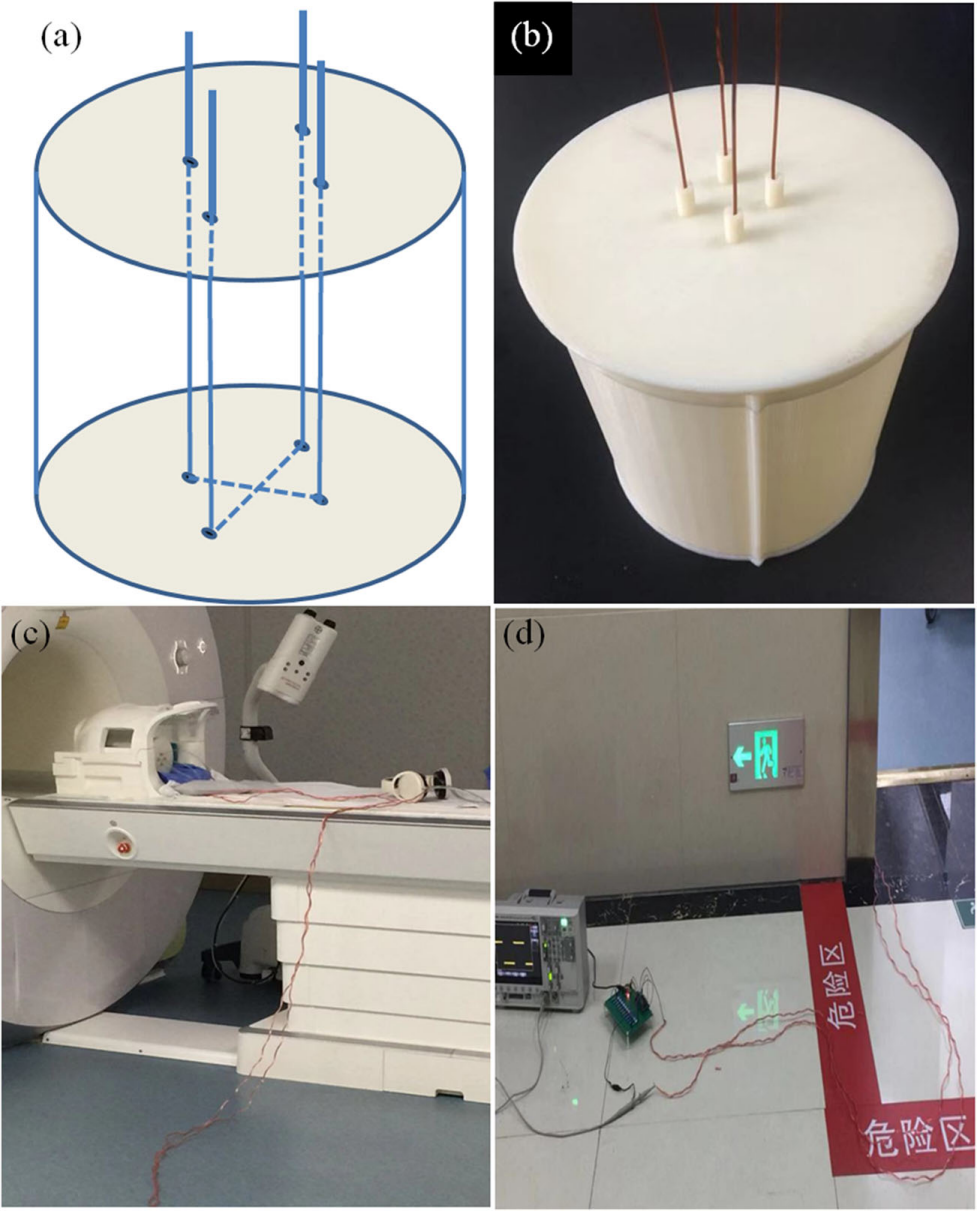
**a** A schematic diagram of the internal structure of the phantom. **b** A photograph of the phantom. **c** The phantom was placed in the dedicated head-neck coil; **d** the stimulation circuit was placed outside the shield room

A polymethylacrylate cylinder tube with an inner diameter of 14 cm and a height of 15 cm was created using a 3D printer with a wall thickness of 1 mm. Four small holes of 1 mm in diameter were punched on the upper and lower sides of the cylinder, with each hole having a pitch of 2.5 cm. Enameled copper wires of 1-mm diameter were passed through the positioning holes. The agarose gel (31.5 g of agarose powder, 21.0 g of sodium chloride, and 4.2 ml of gadolinium diethylene-triamine pentaacetic acid (Gd-DTPA) dissolved in 2100 ml of water) was then poured into the phantom before it was finally packaged (Fig. 1a, b).

### Stimulation circuit

**Fig. 2:**
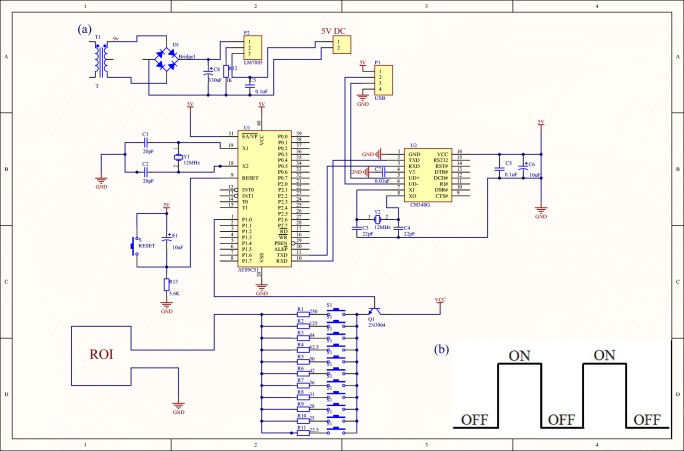
(a) A schematic diagram of the stimulation circuit; (b) the mode of stimulation circuit output stimulation

Based on the 51 single-chip microcomputer, a stimulation circuit capable of outputting a square wave was designed (Fig. 2). The size and frequency of the generated circuit can be adjusted. The voltage could be adjusted within the range of 0–5 V and the current can be varied within 0–240 mA, while the frequency can reach 500 Hz. Therefore, different BOLD stimulation scenarios could be simulated. As illustrated in Fig. 1 c and d, the stimulation circuit was placed outside the shield room and connected to the phantom in the dedicated head-neck coil via a copper wire, to avoid direct effects of the magnetic field and RF pulses on the stimulation circuit.

### Theory

For a voxel at a certain distance from a wire, the MR signal magnitude depends on its distance from the wire and the current level in the wire (including the current direction). If the wire is thin and straight enough and its radius is a, then the current-induced magnetic field can be expressed as follows:

1 $$ \mathbf{B}=\frac{\mu_0I}{2\pi {a}^2}r\kern4.25em \left(\mathrm{inside}\ \mathrm{the}\ \mathrm{wire}\right) $$

2 $$ \mathbf{B}=\frac{\mu_{Gel}I}{2\pi r}\kern4.5em \left(\mathrm{outside}\ \mathrm{the}\ \mathrm{wire}\right) $$

where *r* denotes the distance from a voxel to wire center. *μ*_0_ denotes the permeability of vacuum. *μ*_*Gel*_ indicates the relative permeability of agarose gel. According to Eq. 1 and Eq. 2, it can be inferred that the larger the current (*I*) is, the larger the induced magnetic field (**B**). The closer voxels are located inside the wire, the smaller the induced magnetic field. When voxels are located outside the wire, the farther they are from the center, the smaller the induced magnetic field. The maximum magnetic field is induced on the surface of copper wire when these conditions are reversed.

The current direction is parallel or antiparallel to the main magnetic field (**B**_0_), and the induced magnetic field produced by the current is always perpendicular to the **B**_0_ according to right hand grip rule.

### Scanning protocol

**Fig. 3:**
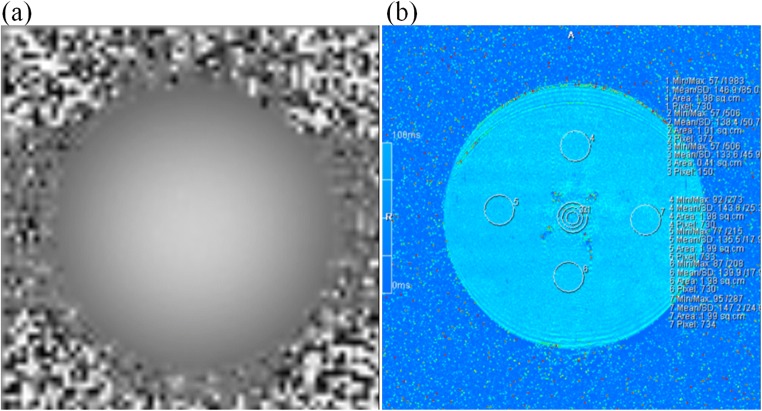
**a** The field mapping; **b** the T2* mapping of the phantom center

Field map scanning and fMRI scanning were performed on three different MR scanners (GE Signa HD 1.5 T, Siemens Verio 3.0 T, and Siemens Skyra 3.0 T). T1 Mapping scan using double-flip-angle technology was acquired with the repetition time (TR) of 15 ms, echo time (TE) of 2.08 ms, flip angle 1 (5°), flip angle 2 (26°), and 30 slices. 5-echo technology was employed in both T2* mapping and T2 mapping. The parameters were as follows: TR = 789 ms, TE1-TE5 (4.36 ms, 11.90 ms, 19.44 ms, 26.98 ms, 34.52 ms), 20 slices; and TR = 1511 ms, TE1-TE5 (13.8 ms, 27.6 ms, 41.4 ms, 55.2 ms, 69.0 ms), 20 slices, respectively. The following parameters for all mapping charts were consistent: FOV = 200 mm × 200 mm, the slice thickness is 3 mm with a 0.6 mm gap, NEX = 1, matrix size = 384 × 384 (Fig. 3).

The single-shot echo planar imaging (EPI) was used in fMRI scan sequence, and the parameters were set as follows: TR = 2000 ms, TE = 30 ms, Flip Angle = 90°, 11 slices, the slice thickness = 5 mm, without gap, interval scanning, FOV = 240 × 240 mm, Matrix = 64 × 64, 100 scans.

The fMRI block design: baseline (no current in the circuit), active state (8, 12, 24, 48 mA). The scans followed the paradigm of alternative baseline-active states, each of which lasted 10 s. Only one copper wire in the phantom was connected to the stimulus circuit. As a comparison, the other copper wire was not introduced into the stimulus circuit. To detect the consistency of stimuli signals, the exams were performed twice with the same parameters. Data analysis was performed using Matlab 2013b (The MathWorks, Inc., Natick, MA, USA).

### Evaluation program and analysis parameters

The average signal intensity of voxels in 100 successive images was obtained. Static space noise was measured according to the National Electrical Manufacturer’s Association (NEMA) [8]. Even and odd signal in the image were summed, and the difference between them was the static spatial noise.

3 $$ Static\ Spatial\ Noise=\left| Image\ {Numbenr}_{odd}- Image\ {Numbenr}_{even}\right| $$

The standard deviation of the image signal values for each voxel at 100 time points is temporal fluctuation noise (TFN).

The ratio of the mean signal value of the ROI (9 pixel) in the center region of the phantom to its fluctuating noise was defined as the temporal fluctuation noise ratio (TFNR).

At baseline state, the mean signal intensity and static space noises of large ROIs (9 pixels) composed of 100 successive images in the same layer were used to evaluate the signal-to-noise ratio (SNR).

4 $$ \mathrm{SNR}={\mathrm{Signal}}_{\mathrm{ROI}}/\sqrt{{\mathrm{Noise}}_{\mathrm{summary}}/100\mathrm{time}\ \mathrm{point}} $$

Also, at baseline state, the mean signal intensity of large ROI (9 pixel) composed of 100 successive images and the mean background signals from each side (upper, lower, left and right) of the peripheral phantom were obtained. The ghosting ratio was computed as follows:

5 $$ \mathrm{Ghosting}\ \mathrm{Ratio}=\raisebox{1ex}{$\left|\left({\mathrm{ROI}}_{\mathrm{up}}+{\mathrm{ROI}}_{\mathrm{down}}\right)-\left({\mathrm{ROI}}_{\mathrm{right}}+{\mathrm{ROI}}_{\mathrm{left}}\right)\right|$}\!\left/ \!\raisebox{-1ex}{$2\times {ROI}_{center}$}\right. $$

The mean signal value for large ROI (9 pixels) in the intermediate layer of the phantom (copper wire in the ROI) was extracted to obtain a time-signal intensity curve. The mean value of signals with the most obvious change at continuous time points was denoted as “Active,” and the average of basal signal values was defined as “Baseline.” The difference between them was divided by baseline and multiplied by 100% to obtain the percent signal change (PSC).

6 $$ \mathrm{PSC}=\frac{\left|\mathrm{Active}-\mathrm{Baseline}\right|}{\mathrm{Baseline}}\times 100\% $$

### Sensitivity

Sensitivity was defined as detection efficiency of a stimulus of an identical frequency through 100 time points. The respective stimulation sensitivities were obtained according to Eq. 7.

7 $$ \mathrm{Sensitivity}=\left(\frac{{\mathrm{N}}_{\mathrm{Actual}}}{{\mathrm{N}}_{\mathrm{Theoretical}}}\right)\times 100\% $$

where the *N*_Actural_ denotes the number of stimuli actually detected, and *N*_Theoretical_ denotes the theoretical number of stimuli.

### Repeatability

To assess the phantom signal stand-alone consistency, three MR scanners were employed repeatedly with the same protocol, and then their stimuli detection efficiency was calculated.

### Active jamming analysis

Periodic current was introduced into the phantom to simulate the task stimulation. To better analyze active interference, we shortened the stimulation period to 12 ms, in which the stimulation lasted for 6 ms. In addition, the radio frequency excitation and gradient switching generated induced current in two pairs of coils. The stimulated and induced currents were monitored by an oscilloscope and detected in real time.

### Statistical parameter map processing

Raw data obtained by different scanners were imported into statistical parameter map (SPM) for preprocessing, such as slice timing and motion correction. The first-order analysis of a single subject was carried out. Then *t*-value maps of the phantom were obtained by GLM analysis.

## Results

### Phantom properties

In conditions of no power, 7 regions of interest (ROIs) were selected through the field map, T1 and T2*, T2 mapping scanning. These ROIs were selected from both center of the phantom and regions around it. The areas of ROI 1, 2, and 3 were gradually increased while those of ROI 3–7 were set at 3 cm^2^. The signal distribution in the phantom was observed and the T1, T2, and T2* values of the central and peripheral regions of the phantom are shown in Fig. 3 and Table 1. The mapping diagram revealed that the four wires carried equal signals, and the average T1 value was significantly lower than that of the peripheral region, while the signals obtained from the peripheral region were identical.

**Table 1:** The relaxation time in the center and peripheral regions of the phantom measured at intermediate level

	T1 (ms)	T2 (ms)	T2* (ms)
Center	437.2 ± 51.7	372.4 ± 939.1	121.2 ± 37
Peripheral	1409.8 ± 137.7	292.5 ± 8.0	121.3 ± 13.4

### Signal stability of agarose in the phantom

**Fig. 4:**
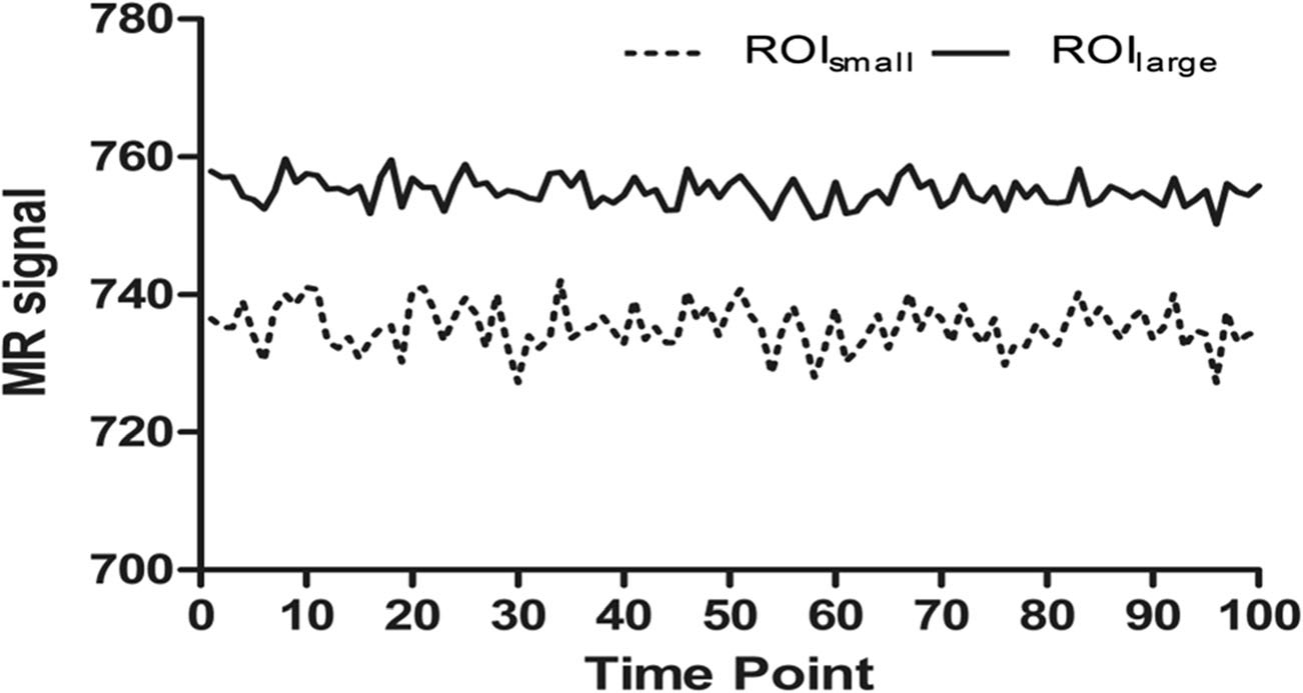
Signal change in the central region of the phantom at baseline state. Solid line indicates the average signal in the ROI of 9 pixels, whereas dotted line indicates the average signal in ROI of 4 pixels

Two ROIs (9 pixels, 4 pixels) from the center of an image acquired using baseline parameters were selected. Analysis of the signal from the central region indicated that it was stable, with an average signal strength fluctuation within 1% and a signal range variation of less than 10 (Fig. 4, Table 2), highlighting the feasibility of using this strategy in subsequent BOLD analysis.

**Table 2:** Indicators of phantom system at baseline state

Pixels’ number	Signal	Static spatial noise	Temporal fluctuation noise	SFNR	SNR	Ghosting ratio (%)
4	735.2	0.1	3.234	227.33	2326.58	0.39
9	755	0.1	1.996	378.26	2389.24	0.38

### Motion analysis of the phantom in magnet

**Fig. 5:**
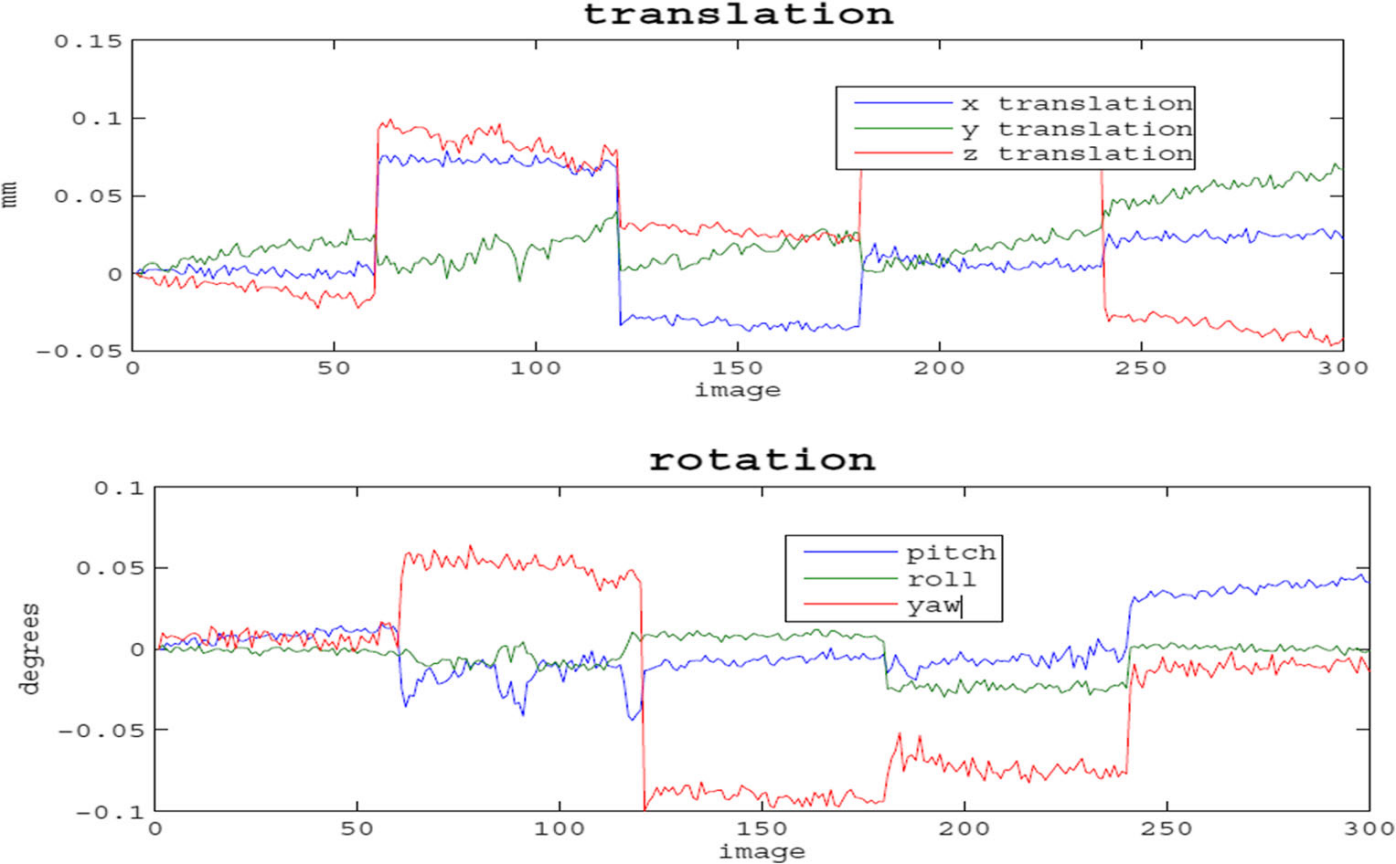
The translational and rotational motion of the phantom during scanning

Further analysis of motion correction revealed that the translation and rotation of phantom was not obvious as phantom translation did not exceed 0.1 mm while rotation was below 0.1 degrees (Fig. 5).

### Characteristic of the phantom under stimulation

**Fig. 6:**
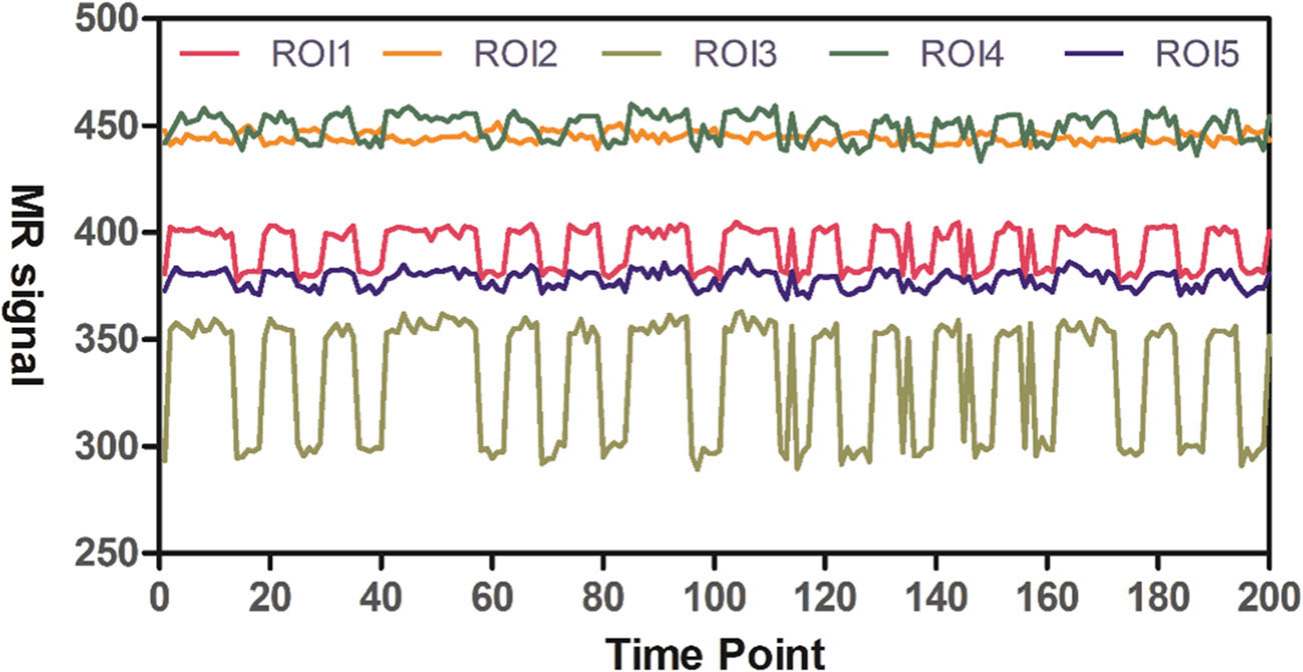
The time-signal intensity curve for different ROI at the same current level

Various indexes of the phantom system at baseline state are shown in Table 2. The sensitivity of the measurements to detect BOLD signals depended on the signal-to-noise ratio (SNR). A 24-mA current was used in the analyses of the different ROIs to demonstrate the time-signal intensity curve within the same layer of the phantom (Fig. 6). Distinct intensity ranges (amplitudes), with highly consistent waveforms, were obtained for different ROIs at both baseline and stimulated states. Percent signal change (PSC) for the aforementioned ROIs was evaluated using different currents (Table 3).

**Table 3:** The PSC of the ROI (9 pixels) in the same slice at different current level

	PSC (%)	I (mA)			
		8	12	24	48
Manufacturer	Siemens skyra 3.0 T	0.61	0.67	1.26	3.75
	Siemens verio 3.0 T	0.69	1.28	2.20	7.94
	GE signa HD 1.5 T	4.11	7.07	21.07	26.16

Among them, the PSC of the second column was significantly different from the other ones, where smaller difference was found in the other groups (Table 3). While the amplitude of each activation was identical and stable, evaluation of PSC was necessitated by the presence of peaks in the active segment, which also caused a higher average signal intensity. PSCs in the same layer were found to vary from 1% to more than 10%, thus covering the variation range of BOLD signal in the human brain. The stimulation amplitudes at different time points in an individual curve were relatively identical, and while the shapes of different curves representing the ROIs were similar. It should be considered that the baseline signal is proportional to the size of ROI. Because sharp peaks occasionally appeared in these curves, this might affect the calculation of PSC, thereby causing errors.

### Phantom signal stand-alone consistency

**Fig. 7:**
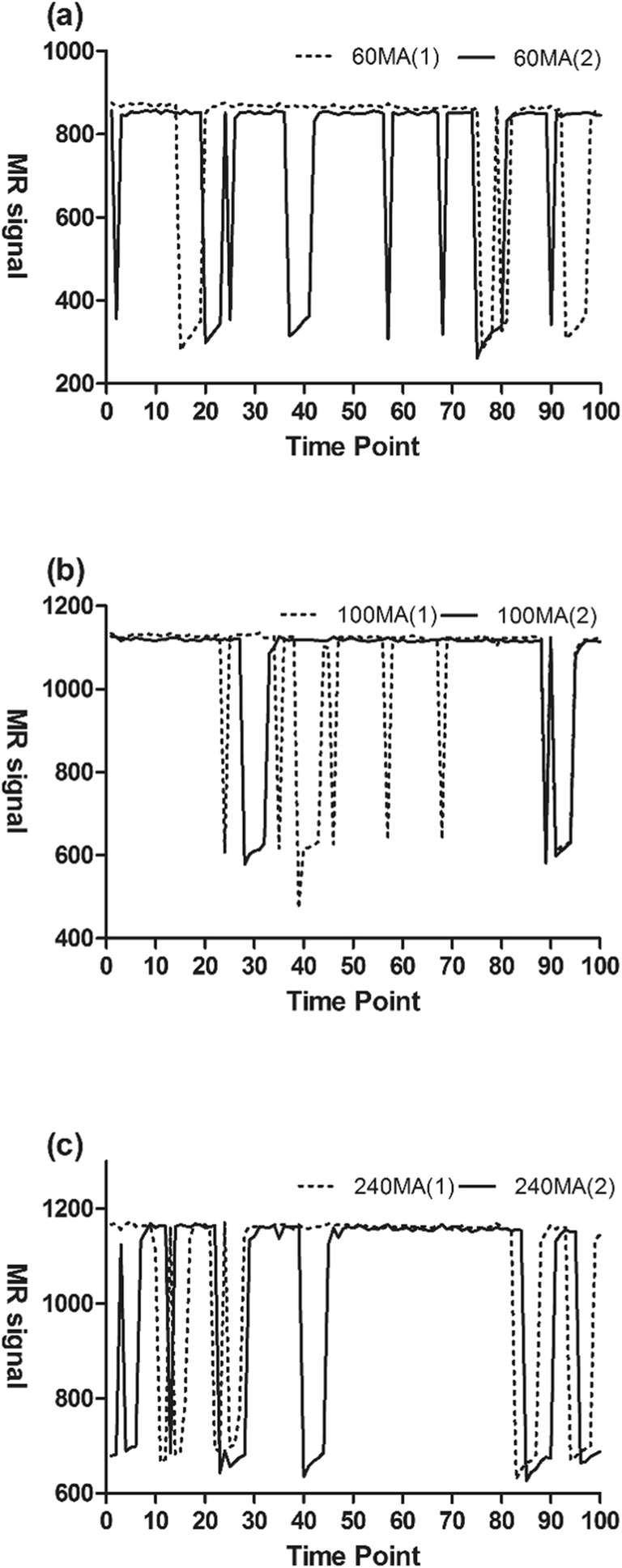
Repeatability results for different current levels. **a** Repeated scanning at 60-mA current level; **b** repeated scanning at 100-mA current level; **c** repeated scanning at 240-mA current level (Siemens skyra 3.0 T)

In order to compute the number of effective stimuli per 100 scanning events, we repeated the scanning on the same scanner. All three scanners exhibited good reproducibility but results obtained from the Siemens Skyra 3.0 T scanner are shown (Fig. 7).

### Sensitivity measurements

**Fig. 8:**
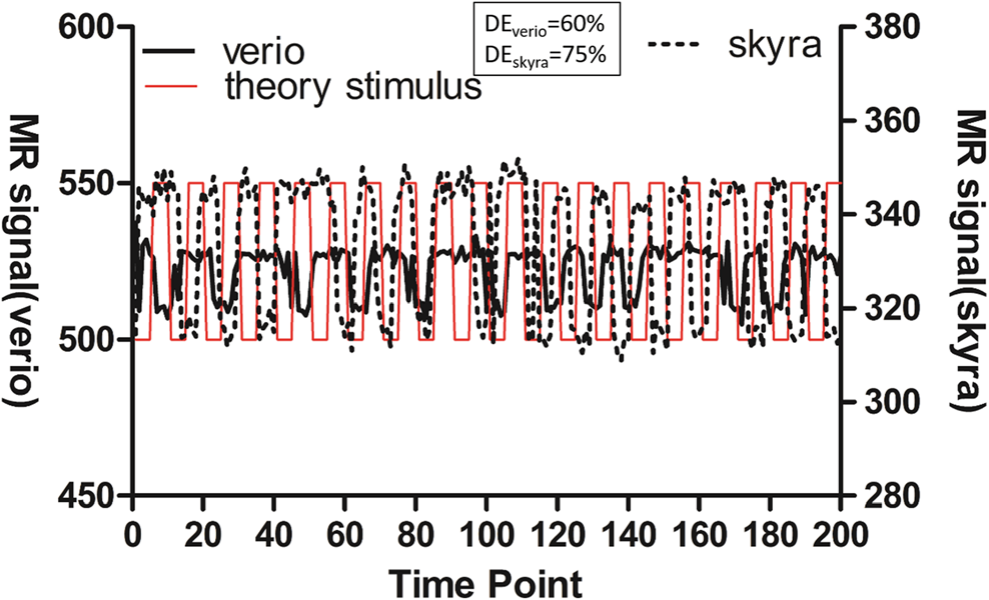
Number of stimuli detected by different scanners (solid line for Siemens verio 3.0 T, dashed line for Siemens skyra 3.0 T, and solid red line for theoretical stimulus, DE denotes detection efficiency)

Here, we define sensitivity as the detection efficiency of stimuli of an identical frequency through 200 time points. To do this, we extracted the time-signal intensity curves of the phantom from the scanning images obtained by two MR scanners, Siemens Verio and Siemens Skyra. The stimuli that were effectively for the two scanners was 60% and 75%, respectively (Fig. 8).

### Active jamming analysis

**Fig. 9:**
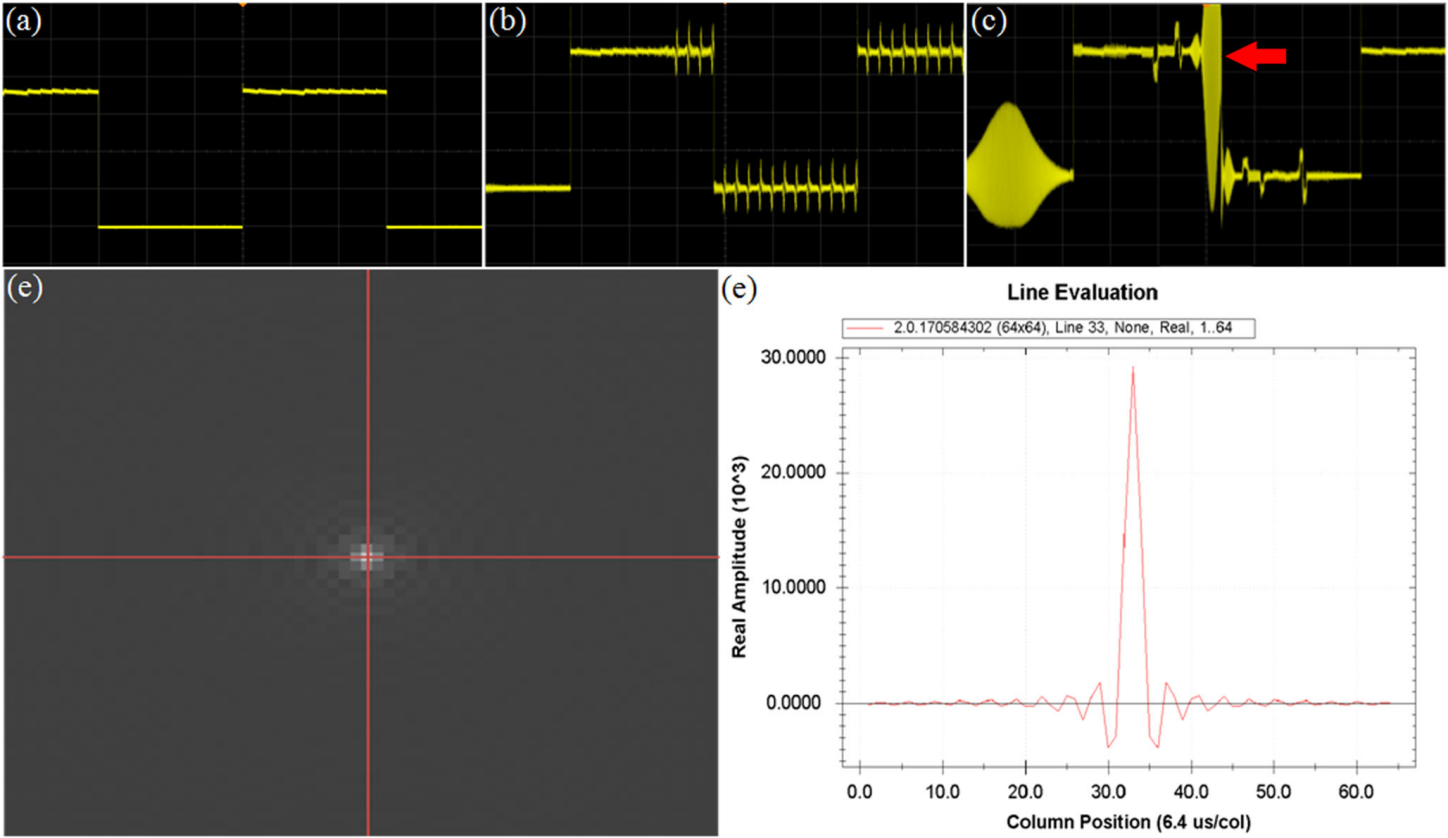
Active jamming analysis. **a** The stimulation circuit was connected to the phantom but was not scanned. The signal was stable, without spurious peak. **b** The stimulation circuit was connected to the phantom scanned, creating regular and high-frequency clutter in the circuit. **c** The oscilloscope was adjusted to amplify the waveform during the scanning process. Most sharp parts of the noise waveform did not exceed the range from the ground state to the active state, but occasionally, a sharp wave exceeded the range (red arrow). **d** The k-space map obtained via Fourier transform. **e** Amplitude map of each point on the horizontal red line in the center of k-space

A periodic current was introduced into the phantom to simulate the task stimulation. At the same time, radio frequency excitation and gradient switching was generated to induce current in two pairs of coils. Both the stimulated and induced currents were monitored by an oscilloscope and visualized in real time. From this analysis, it emerged that the frequency of current stimulation was low, whereas a relatively high-frequency waveform was observed during the scanning. This could be easily captured on the oscilloscope even if both their periods were stable (Fig. 9). Additionally, the amplitude of induced current generated by the circuit was smaller than the stimulus current. In order to obtain k-space, images were chosen at different time points, during which no abnormally high noise was present. This analysis showed that the maximum amplitude of k-space is almost the same at different points, and the variation amplitude is less than 0.001. The actual detected stimulus time was consistent with the theoretical stimulation time, and no other periodic signals with the same stimulation frequency were found from the scanning.

### Statistical parameter map results

**Fig. 10:**
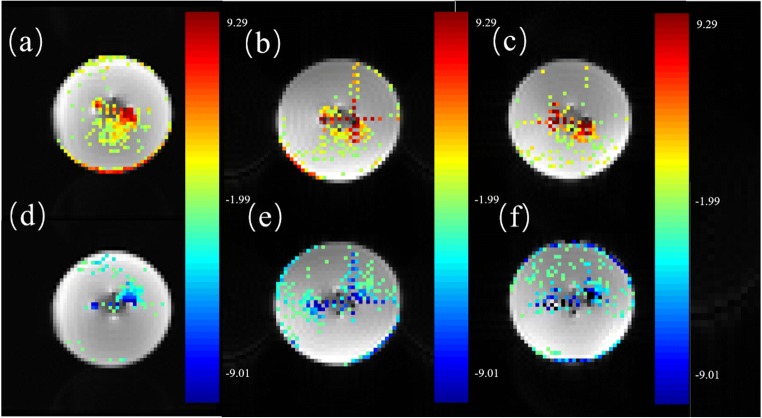
t-Maps from SPM obtained by different MR scanners under the same stimulus (*p* = 0.05, cluster size ≥ 5). (a, d) t-map obtained by GE signa HD 1.5 T; (b, e) t-map obtained by Siemens verio 3.0 T; (c, f) t-map obtained by Siemens skyra 3.0 T. The upper rows (a, b, c) were the positive activation region, and the arrows below (d, e, f) represent the negative activation region. The positive and negative activation regions were in opposite directions and appeared in the direction of the readout gradient

Finally, we subjected the raw data to slice timing and head motion correction using SPM while the t-map of the phantom intermediate layer was obtained through GLM analysis (Fig. 10). Comparing different scanning instruments under the same stimulation and scanning conditions revealed that activation areas obtained by Siemens Skyra 3.0 T were largest, while the GE Signa HD 1.5 T produced the smallest activation area. However, their activation areas were obtained within similar locations.

## Discussion

In this study, uniformity of local magnetic field in gel water molecules was altered to simulate the BOLD signal which was then used for quality control assessment of signal detection efficiency in fMRI. The BOLD signal obtained by scanning the phantom was stable. This task-based imaging system was used for obtaining signals of controllable change range. In addition to computing SNR, ghost ratio, and SFNR, by using this method, we can carry out different enhancement levels in the same layer, and evaluated the sensitivity of different scanners to BOLD signals, besides computing SNR, signal ghost ratio, and SFNR.

In order to effectively eliminate the influence of the active signal on the imaging device, we applied insulated enamel to the bare copper wire so as to avoid direct contact between wires and the gels of the phantom. Because copper is a diamagnetic material, it effectively reduces artifacts, and thus increases the area available for analyses [14, 15]. The stimulation circuit was connected to the phantom and placed out of the shield room, after which a stable stimulation waveform was outputted from the stimulating circuit without scanning. Sharp interference waveforms of high frequency appeared on the oscilloscope during scanning. The emission frequencies of the interference waves were consistent with radio frequency (RF). Previously published studies suggest that this might be because RF excitation and the fast switching of the gradient magnetic field produces an induced current of high frequency in the copper wires of the phantom [16]. However, in our analysis, the induced current and stimulated current differed significantly in both waveform and frequency with little overlap between them. This phenomenon enabled their clear resolution. We therefore considered exploiting the difference in frequency to limit the high-frequency noise information through low-pass filtering [17]. Our experiments showed that the phantom signal was of superior quality while artifacts around the copper wires were limited. When electric current passed through the copper wire, an induced magnetic field was generated around the wires. Effects of the induced magnetic field on voxel were decreased in parallel with an augmentation of the distance between voxel and copper wires, thereby affecting various ROIs in the same layer differently. PSC analysis of the phantom showed that it ranges from 1 to 10%, and covered the range of variation of the human brain BOLD signal. The frequency of stimulation created by the blocking experiment was low (0.05 Hz), and the subsequent analysis showed a significant decrease in the signal value caused by the stimulus. However, occasional peak and abnormal values appeared during the whole scanning. These abnormal values might have resulted from RF excitation and gradient magnetic field switching since the high-frequency sharp waveforms observed in the oscilloscope were quite consistent with RF.

In our analysis of the signal obtained at the center and periphery of the phantom, we found that the signal from the phantom was of good uniformity, especially in the central region. This analysis showed that its pixel value deviated from the average value within less than 1% of the average signal intensity. Therefore, the central area could be used as the main area for function analysis on which calculation of the signal-to-noise ratio, contrast-to-noise ratio, and ghost-to-signal ratio could be carried out. Our fMRI phantom fundamentally differs from the human brain because it does not experience physiological process including breathing, pulse, and head movement, which interfere with human BOLD signal [9]. The translational and rotational motion of the phantom during the entire scan did not exceed 3% of the voxel size. Such small movements might result from the vibration of the patient table or the machine itself.

Taken together, our data shows that the BOLD signal based on voxel level can be directly performed so as to avoid amplification and partial losses to the original signal occasioned by data preprocessing. This would bring the data closer to the true value [4, 18]. Detection efficiency of the same scanner was highly reproducible after repeatedly scanning the phantom with different current magnitudes and magnetic field strengths [15]. Using the same stimulation mode, the PSC of each stimulus detected by the same scanner was close. This reflected the intensity of each stimulus, reduced contingency, and improved the stability and reproducibility of the phantom. Considering that the phantom’s stimulation circuit was different from the human brain, which can be adapted to stimuli [19], our analysis attained the scanner stability and sensitivity to stimulus by scanning the phantom repeatedly using the same stimulus mode.

A limitation of traditional passive fMRI phantoms is that they simulate the BOLD signal changes by taking advantage of the difference in T1 and T2 relaxation time caused by different concentrations of the same material [12, 20], which does not simulate true stimuli. In addition, multiple processes require manual operation, introducing a high number of experimental variables. Relative to the Smartphantom [9], our approach enhanced the area available for analysis in the phantom. In addition, our system was found to be compatible with different scanning equipment. Moreover, the versatility of our approach means that the duration and intensity of the stimulus can be accurately adjusted to obtain the optimal stimulus scheme.

While our system presented multiple advantages over established methods, it has some limitations. Firstly, the agarose gel used in our study was not suitable for long-term preservation and was prone to decomposition. This led to air separation around the copper wires which in turn adversely affected image quality and data analysis. To address this limitation, we are currently optimizing the phantom’s physical properties and are considering the use of different materials, such as PVC or HDPE. Additionally, in order to realize the quality control for software analysis process of fMRI, the stimulation of BOLD signal will be combined with task design, and display activated areas with SPM Software.

## Conclusions

The phantom we designed for fMRI quantitative quality control is easy to operate and highly compatible with different scanners, providing reliable data obtained through a simple and straightforward data processing pipeline. These strengths make the proposed phantom suitable for daily quality control applications for fMRI analyses.

## Abbreviations

BOLD: Blood-oxygen-level-dependent
fMRI: Functional magnetic resonance imaging
SNR: Signal-to-noise ratio
ROI: Region of interest
PSC: Percent signal change
Gd-DTPA: Gadolinium-diethylenetriamine pentaacetic acid
FOV: Field of view
TFN: Temporal fluctuation noise
TFNR: Temporal fluctuation noise ratio
SPM: Statistical parameter map
